# Public Roads as Places of Interspecies Conflict: A Study of Horse-Human Interactions on UK Roads and Impacts on Equine Exercise

**DOI:** 10.3390/ani11041072

**Published:** 2021-04-09

**Authors:** Danica Pollard, Tamzin Furtado

**Affiliations:** 1 Safety Department, The British Horse Society, Abbey Park, Stareton, Kenilworth, Warwickshire CV8 2XZ, UK; 2 Institute of Infection, Veterinary and Ecological Sciences, University of Liverpool, Leahurst Campus, Neston CH64 7TE, UK; tfurtado@liverpool.ac.uk

**Keywords:** horse riding, horse-related injuries, road use, road traffic accidents, road safety, vulnerable road users

## Abstract

**Simple Summary:**

The risk posed by traffic likely impacts equestrians’ decisions around road use, potentially limiting or preventing exercise sessions. This study identifies how frequently equestrians use roads and what impacts their ability to do so. Over 6000 UK equestrians completed an online questionnaire about their exercise behaviours, road use and experiences of road-related incidents. Most equestrians use roads regularly. In the previous year, 68% of equestrians experienced a near-miss and 6% an injury-causing incident. Our results found that regional differences in road use and near-miss experiences coincided with off-road route availability. Road use was associated with the proximity of off-road routes, and road-using equestrians covered longer distances. Near-misses were associated with increasing frequency of weekly road use. Younger equestrians were more likely to use roads, but also to experience near-misses. Injury-causing incidents were associated with increasing road-use anxiety or ceasing to use roads (due to strong feelings of danger, compromised safety and conflict with other road users), the proximity of off-road routes, having a near-miss and riding while leading a ridden horse; often a child. Targeted campaigns encouraging responsible road use, better off-road access and inclusion of equestrians in planning and development initiatives would create safer equestrian spaces, particularly for young people.

**Abstract:**

Real or perceived traffic risk is a significant barrier to walking and cycling. To understand whether similar barriers influence equestrians, this study obtained exercise behaviours, road use and experiences of road-related incidents from UK equestrians (*n* = 6390) via an online questionnaire. Multivariable logistic regression models were used to identify factors associated with road use and experiencing a near-miss or injury-causing incident in the previous year. Content analysis identified themes around equestrians’ decisions not to use roads. Our results show that most equestrians (84%) use roads at least once weekly, and in the previous year, 67.7% had a near-miss and 6.1% an injury-causing incident. Road use differs regionally, with exercise type and off-road route availability. Road-using equestrians covered greater daily distances and were younger. However, younger equestrians were at higher risk of near-misses. Respondents’ decisions not to use roads were based on individualised risk assessments arising from: the road itself, perceptions of other road users, the individual horse and the handler’s own emotional management. Roads were perceived as extremely dangerous places with potentially high conflict risk. Injury-causing incidents were associated with increasing road-use anxiety or ceasing to use roads, the proximity of off-road routes, having a near-miss and type of road use. Targeted road-safety campaigns and improved off-road access would create safer equestrian spaces.

## 1. Introduction

Horse-related road incidents in the United Kingdom (UK) are common; 60.3% of equestrians surveyed in the UK (*n* = 257/426) reported having a near-miss on the road in the previous year [1]. Equestrians surveyed in Devon (*n* = 1976), a county in the South West region of England, report that 79.1% had experienced a near-miss, 15.6% a collision with a vehicle, and 7.7% had sustained an injury when using roads with their horses [2]. Being passed too closely by a vehicle driver was one of the single most significant contributors to collision risk between a vehicle and an equestrian and their horse, while collisions and speeding were associated with a higher risk of fatal injury to the horse [3]. The severity of injury to a horse in a road-related incident was also associated with significant injury to the equestrian; a fatal injury to a horse was almost 12 times as likely to result in severe to fatal injury to the equestrian.

Equestrians are often considered low-priority road users by transport policymakers [4], despite millions of people in the UK being involved in the equestrian sector [5]. There remains a lack of recognition that their status as vulnerable road users is amplified because horses are not vehicles but, alongside their human partners, are vulnerable road users in their own right. Although research into equestrian road safety has gained some traction over recent years [2,3,4,6,7,8], it is still a relatively under-researched topic. To better understand the impact that road safety has on equestrians, it is important to identify how frequently they use roads and what affects their ability to do so.

Published data around equestrian road use in the UK is sparse. The typical distances travelled by horse riders in Devon, across seasons and days of the week, and the types of roads ridden on are available [2]. However, similar information for the wider UK equestrian population is currently lacking. Additionally, it is imperative to gain a better understanding of how perceptions of risk and previous experiences on roads may impact the ability of equestrians to exercise their horses. Research on the impacts of traffic on other vulnerable road user groups has identified an inverse relationship between traffic volume and/or speed and levels of walking and cycling; whether the danger posed by traffic is real or perceived [9]. Roads with high volumes of traffic and/or fast-moving vehicles present physical and psychological barriers to pedestrians and have been shown to limit walking trips [9,10,11] and affect general wellbeing [12]. Perceived traffic risk is a major barrier to cycling, particularly for potential or occasional cyclists and although experiences of both near-misses and collisions contributed to risk perception, near-misses more strongly influenced perceived traffic risk [13]. It is highly likely that similar perceptions of road conditions and prior experiences influence equestrians’ decisions around using roads and potentially contribute to shorter exercise sessions or a reduced motivation to exercise their horses.

For this study, we aimed to employ a mixed-methods approach to obtain information from UK equestrians about how frequently, and in what capacity, they used roads with their horses. Additionally, we aimed to investigate what may influence their decisions to use roads and what impact this may have on their combined exercise habits. The main objectives were to:

1. Describe the activity of equestrians when exercising their horses, particularly with regards to their use of off-road routes and roads
2. Identify factors associated with road use by equestrians
3. Identify themes from equestrians’ comments as to their decisions to not use roads with their horses
4. Describe road-related incident experiences and views around incident reporting
5. Identify factors associated with equestrians having had a near-miss or injury-causing incident, while using roads with their horse in the previous year
6. Provide a solid evidence base that can assist road safety stakeholders to invest in targeted campaigns that encourage responsible behaviour around horses on the road, thereby improving road safety for all road users.

## 2. Materials and Methods

An online questionnaire was available between 1 October and 15 November 2020 for completion by equestrians based in England, Scotland, Wales and Northern Ireland (Questionnaire S1). Owners, loaners and sharers of horses, ponies, donkeys and mules (collectively referred to as horses) were invited to participate.

Participants were asked to provide information via closed-ended questions about their regular activity with their horses, including:

- Any structured exercise they did with their horses (exercise type, weekly frequency, average time and duration during weekdays and the weekend);
- Use of off-road routes when exercising their horses (frequency of use, availability and proximity of off-road routes);
- Use of public roads when exercising their horses (exercise type when on the road, weekly frequency, proportion of road vs off-road routes used).

Participants were asked whether, in the previous year, they had experienced any incidents while exercising their horse on the road, including incidents that made them or their horses feel unsafe, near-misses or incidents resulting in injury to them or their horse. They were also asked to rate their level of anxiety when using roads with their horses (“I don’t feel at all anxious” to “I feel extremely anxious”). Awareness of the British Horse Society (BHS) Horse Incidents reporting website [14] was assessed by asking participants whether they knew about it and to rate, on a scale of 0 (not at all likely) to 10 (very likely), how likely they would be to report an incident. Three open-ended questions explored participants’ opinions of what would make them more or less likely to report a horse-related road incident to the BHS incident reporting website and for those that said they did not use roads with their horses, allowed them to express in their own words, the reasons behind this.

The link to the questionnaire was shared in various equestrian groups on social media, both by the BHS, but also by other large UK-based equestrian welfare organisations, and included in the BHS members’ e-newsletter. People completing the questionnaire were also encouraged to share it with equestrian friends and family. Informed consent was obtained from all participants, and only those wanting to participate in further equestrian road safety research volunteered their contact details (email address); otherwise, the questionnaire was completed anonymously.

### 2.1. Quantitative Data Analysis

Questionnaire data were exported into a Microsoft Excel (Office 365, Microsoft Corporation, Redmond, WA, USA) spreadsheet for initial data cleaning. Coding and quantitative data analyses were conducted in Stata (IC v. 13.0, StataCorp LP, College Station, TX, USA). Categorical data were described as proportions (%) with 95% confidence intervals (CI), and ordinal data were described as medians with interquartile range (IQR). Relationships between categorical variables were assessed using the Chi-square or Fisher’s exact test. Three ordinary logistic regression models were built to identify factors associated with near-misses and injury-causing incidents while using roads, as well as general road use by equestrians by calculating odds ratios (OR) and corresponding 95% CIs.

The first binary outcome variable used was whether the participant used roads with their horses (1) or did not (0). Univariable logistic regression models were used to identify factors associated with higher odds of equestrians using roads with their horses. Explanatory variables tested included the participant’s country and region, years of equine experience, age, approximate distance covered and time spent exercising their horses, distance to nearest off-road route, how well off-road routes connected, the weekly frequency of any regular exercise they do with their horses and the type of exercise they did with their horses in general.

The second binary outcome variable used was whether the participant had experienced a near-miss while using roads with their horse in the previous year (1) or had not (0). The third binary outcome variable used was whether the participant had experienced an injury-causing incident while using roads with their horse in the previous year (1) or had not (0). Univariable logistic regression models were used to identify factors associated with higher odds of having had a near-miss or an injury-causing incident. Explanatory variables tested included the participant’s country and region, years of equine experience, age, weekly frequency of road use, distance to nearest off-road route, how well off-road routes connected, the type of exercise they did with their horses on the road, the proportion of road to off-road routes and their level of anxiety when using roads. Having had a near-miss in the previous year was also included as an explanatory variable in the injury-causing incident model.

Variables where the LRS (likelihood ratio statistic) *p* < 0.25 were taken forward to multivariable modelling. All three multivariable models were built using manual, forward selection with stepwise addition of variables from most to least significant based on their LRS *p*-values. Variables that significantly improved model fit (LRS *p* ≤ 0.05) were retained in the final model. All variables initially tested, but not retained in the final model, were individually forced back in to assess any potential interactions or confounders not previously identified. Responses that had missing data on variables of interest were automatically excluded from the analyses during model building. Categories with a low number of observations were combined with their nearest category, if appropriate (i.e., they shared the same effect), to create a more stable model. For example, the Greater London region had only 20 observations, and in some of the model, it was combined with the South East region. The Hosmer–Lemeshow goodness-of-fit test assessed the fit of the final logistic regression models to the data [15]. A receiver operating characteristic (ROC) curve was additionally estimated to calculate the area under the curve.

### 2.2. Qualitative Data Analysis

The open-ended questions were analysed using a conventional content analysis methodology, as described by Hsieh and Shannon [16]. Conventional content analysis is a useful approach for open-ended survey data when little is known about the target phenomenon, and involves identifying common, descriptive themes which arise in the data, before analysing those themes to create overriding categories, thus providing a more in-depth understanding of the phenomenon [17]. Unlike some other types of content analysis, the conventional content analysis does not attempt to count or quantify qualitative data [16]. This approach was selected in order to provide a method for organising and better understanding participants’ responses, and to support and complement the quantitative data presented in this study. The analysis was undertaken in Microsoft Excel.

First, the authors familiarised themselves with the dataset, making notes of important ideas and themes. Next, each individual response was analysed, with initial categories of meaning being developed according to the component parts of the response. For example, if an item mentioned that the roads near them were dangerous and they could pay for off-road riding, this was considered two themes: perception of roads as being dangerous, and alternative routes being available. Themes were, therefore, developed iteratively, as more and more data were captured. Following this stage, theme contents were checked, items were moved when necessary, theme labels were refined as required, and interconnections examined. At this stage, overriding categories were developed according to the interconnections between themes. This ultimately led to the creation of a set of themes and categories, and ultimately the thematic model shown here.

## 3. Results

In total, 6390 questionnaires were received. Sixty-three questionnaires were from participants who did not own, loan or share a horse, 199 were from participants residing outside of the UK, and 311 were only partially completed. Therefore, 573 questionnaires were excluded, and 5817 questionnaires were available for descriptive and statistical analysis. The denominators for some of the descriptive data differ where specified. 

### 3.1. Participant Demographics

A summary of participant demographics is presented in Table 1. Most of the participants were from England (77.2%), and responses were most frequent from the South West region of England (15.5%), the East of England (14.3%) and the South East of England (13.8%). Approximately half of the participants were in the 45 to 64 year age category (52.5%) and had more than 30 years of equestrian experience (57.8%). Approximately half of the participants (53.4%) held membership with the BHS at the time of completing the questionnaire.

Most of the participants owned or cared for one (40.6%) or two horses (29.5%). Approximately a third of horses were kept at the participant’s home premises or at a ‘Do-it-yourself’ (DIY) livery yard, while 16.0% were kept at a rented private yard or field, or at a yard with assisted livery options (7.8%).

### 3.2. Activity around Structured Equine Exercise

Regular structured exercise was defined as any in-hand, driving or riding activity the participant did with their horse/s at least once per week. Most participants (96.7%, *n* = 5584/5772) reported they regularly exercised one or more of their horses. Participants most frequently reported regularly exercising either one (55.1%, *n* = 2984/5420) or two (29.3%, *n* = 1588) horses.

**Figure 1:**
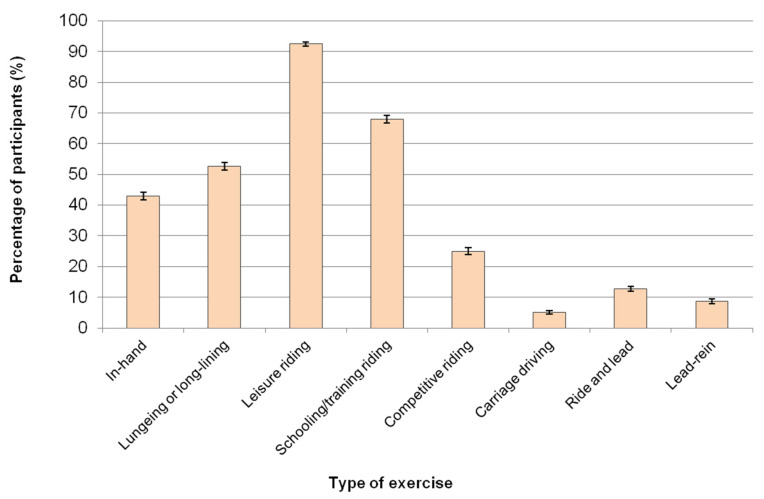
The types of exercise UK equestrians (*n* = 5427) reported doing with their horses at least once a week. Equestrians could select multiple exercise options; therefore, the exercise categories are not mutually exclusive. Error bars represent 95% confidence intervals.

In an average week, 96.0% (*n* = 5212/5427) of participants reported riding their horses. Figure 1 shows the different types of exercise participants reported doing with their horses. Lungeing refers to working a horse in-hand on a circle with a single long rein; long-lining refers to working a horse in-hand with two long reins, ride and lead refers to riding a horse while leading another non-ridden or ridden horse, while lead-rein refers to leading a ridden horse on foot (often a child rider, but could also represent an experienced rider/young horse combination).

**Figure 2:**
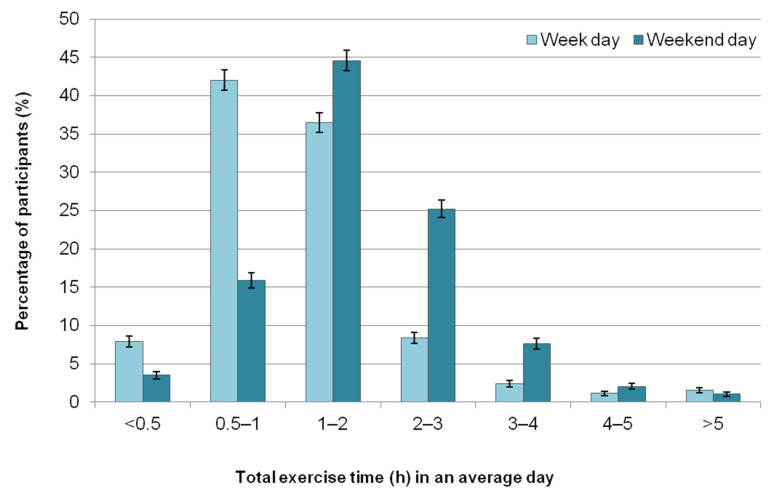
The total time (hours) spent exercising all horses owned/cared for on an average day as reported by UK equestrians (*n* = 5424) for both weekdays (Monday to Friday) and weekend days (Saturday to Sunday). Most participants (84.4%) regularly exercised one or two horses. Error bars represent 95% confidence intervals.

**Figure 3:**
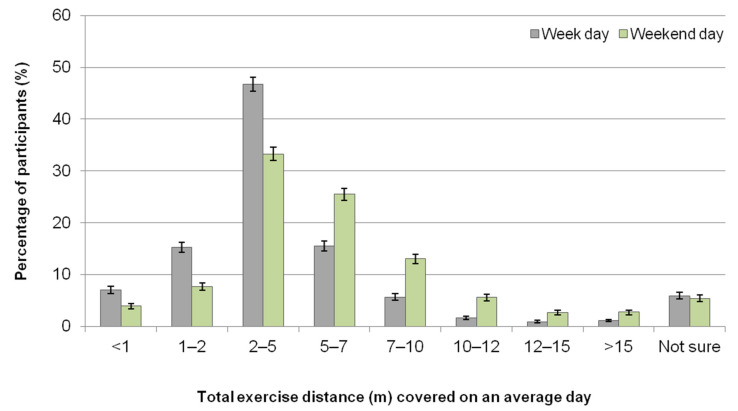
The total distance (miles) covered with all horses owned/cared for on an average day as reported by UK equestrians (*n* = 5423) for both weekdays (Monday to Friday) and weekend days (Saturday to Sunday). Most participants (84.4%) regularly exercised one or two horses. Error bars represent 95% confidence intervals.

Participants most frequently reported exercising their horses between three to five days per week (47.4%, *n* = 2572/5426); 9.6% (*n* = 521) exercised their horses between one to two days per week, while 7.5% (*n* = 408) exercised their horses between six to seven days per week. During an average weekday (Monday to Friday), the total duration of exercise time for all horses owned or cared for was most commonly reported to be between 30 min to two hours (78.6%, *n* = 4261/5425) and most frequently covering a distance of one to seven miles (77.5%, *n* = 4204/5423) (Figure 2 and Figure 3). The total duration of exercise during the average weekend day (Saturday to Sunday) for all horses owned or cared for was most frequently between 30 min to three hours (85.7%, *n* = 4650/5424), usually covering a distance of two to 10 miles (71.8%, *n* = 3896/5423) (Figure 2 and Figure 3).

### 3.3. Activity around Using Off-Road Routes to Exercise Horses

A summary of equestrians’ activity around using off-road routes with their horses is detailed in Table 2. In an average week, most participants used free public or private off-road routes for exercising their horses between one to four days per week (58.4%), although 8.9% said they did not use them at all or used them infrequently, meaning less than once per week (14.0%). Tolled off-road routes (routes where a monthly or annual access fee is paid) were used by 11.5% of the participants, mostly infrequently (48.0%) or one to two days per week (24.1%).

More than half of the participants (53.2%) felt that there were few local off-road routes available to them, with most (33.7%) selecting the “very few” option. Although half of the participants (50.9%) said that the nearest off-road route was within a mile of where their horse was kept, 13.3% said their nearest off-road route was between two to five miles away, and 8.2% said it was more than five miles away. When asked about how well off-road routes connected to each other, 59.2% felt that their local off-road routes were poorly connected, in that they were separated by a considerable amount of road, and only 7.2% felt their off-road routes were very well connected with little to no road separating them.

There was a relationship between the availability of off-road routes and the UK region (*p* < 0.001, *χ*^2^ 282.4), as well as how well the off-road network was connected (*p* < 0.001, *χ*^2^ 223.3). The highest proportion of equestrians reported very few and very poorly connected off-road routes to be available to them in Northern Ireland, Wales, West Midlands and the North West, while the highest proportion of equestrians in Scotland reported they were spoilt for choice when it came to off-road routes, and their routes were very well connected.

### 3.4. Activity around Using Roads to Exercise Horses

**Figure 4:**
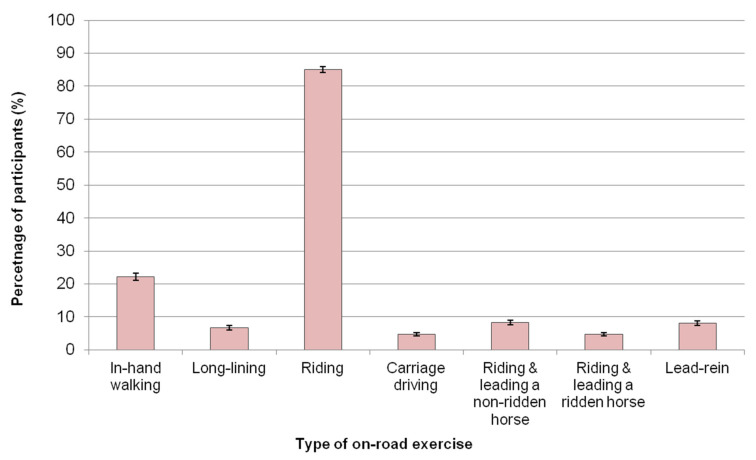
The types of on-road exercise UK equestrians (*n* = 5426) reported doing with their horses at least once a week. Equestrians could select multiple exercise options; therefore, the exercise categories are not mutually exclusive. Error bars represent 95% confidence intervals.

A summary of equestrians’ activity around using roads with their horses is detailed in Table 2. Most participants (69.4%) said that, in an average week, they used or crossed public roads with their horses between one to five days per week and 8.8% used roads on an almost daily basis. Sixteen percent (*n* = 854) said they used roads either infrequently or not at all. Equestrians used roads with their horses in a variety of ways, including for ridden exercise (85.0%, *n* = 4613/5426), for in-hand exercise (22.2%, *n* = 1202) and riding while leading another non-ridden horse (8.2%, *n* = 447) (Figure 4).

When asked about the proportion of road to off-road exercise they did with their horses in an average exercise session, most (28.7%) said they did 80% road to 20% off-road exercise, although a similar proportion (28.5%) said they did 20% road to 80% off-road exercise. However, 9.1% of participants said their average exercise sessions consisted of mostly roads (Table 2).

### 3.5. Contributors to Road Use or Avoidance

Although the majority of participants used roads to exercise their horses either frequently or infrequently, 6.4% (*n* = 346/5426) said they no longer used roads with their horses.

#### 3.5.1. Factors Associated with Road Use

Nine factors were found to be associated with whether equestrians used road to exercise their horses (Table 3). Equestrian road use was associated with: (i) UK region with road use more frequent in the South West (OR 4.4, CI 2.5, 7.5), North West (OR 3.4, CI 1.8, 6.2), Wales (OR 2.5, CI 1.5, 4.3), Yorkshire and The Humber (OR 2.0, CI 1.0, 3.1) and West Midlands (OR 1.8, CI 1.0, 3.1) compared to Scotland; (ii) longer daily distances covered with horses on a weekend day (*p* < 0.001); (iii) the proximity of local off-road routes with road use more likely when off-road routes were between 1 to 2 miles away (OR 2.4, CI 1.4, 4.0) compared to more than five miles away; (iv) how well connected the local off-road network is with road use more likely when the networks were less well connected (*p* < 0.001); (v) equestrians’ age, with road use more likely by younger equestrians (*p* < 0.001); and the types of overall exercise equestrians did with their horses with road use more likely by equestrians who use their horses for (vi) leisure riding (OR 4.3, CI 3.0, 6.0) or (vii) carriage driving (OR 2.9, CI 1.4, 6.1) and road was less likely by equestrians who exercised their horses (viii) in-hand (OR 0.8, CI 0.6, 1.0) or (ix) ridden schooling/training (OR 0.7, CI 0.5, 0.9). Univariable results of the road use model are presented in Table S2.

The Hosmer–Lemeshow goodness-of-fit test (*p* = 0.07, *χ*^2^ 8.51) indicated the logistic regression model described the data well. The area under the ROC curve was 0.81 (CI 0.79, 0.84), suggesting the model had good overall predictive power.

#### 3.5.2. Decisions around Road Use

**Figure 5:**
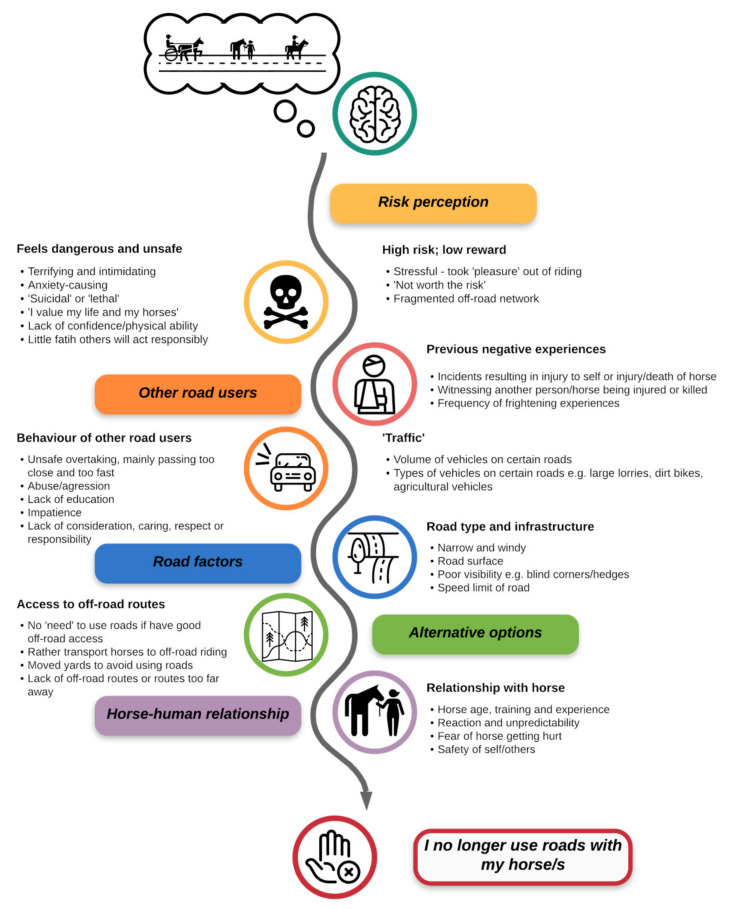
A depiction of the categories and themes which contribute to UK equestrians’ decisions to no longer use roads with their horses, based on each specific horse-human dyad and locale.

Content analysis of equestrians’ comments around decisions to no longer use roads revealed that participants assessed the risk of road use by considering factors intrinsic to the rider-horse dyad, alongside extrinsic factors to do with their perception of the roads themselves, options available for riding, and other road users (Figure 5). Here, we first describe the intrinsic factors which contributed to decisions to no longer use roads, before describing extrinsic factors and the interrelations between the two.

Themes within the intrinsic factors category included the horse-human relationship, participants’ own confidence or ability in managing their horse, their past experience of road use, and the horse’s behaviour (e.g., whether it was inexperienced, or known to dislike traffic). Participants weighed up different elements of these intrinsic factors to make decisions about their safety when on roads based on each specific horse-handler pairing. For example, the following participants suggested that they might use roads with horses that they trusted or had an existing relationship with, but would avoid it with horses whose behaviour suggested they may be less reliable:

*“I avoid using roads with my green mare because I don’t feel it’s safe or fair as her learning experience to be exposed to the level of traffic & speed of vehicles as a starting point.”*

*“My horse was confident on the road but has been scared by drivers approaching too fast. She is now dangerous to hack as a direct result of speeding drivers”*

In these examples, the horse’s behaviour is one of the motivating factors preventing road use. Contrastingly, some respondents suggested that it was their own nerves (regardless of the horse they are paired with) that led them to alter their behaviour to no longer use roads:

*“I owned a horse that was dangerous in traffic, now I am nervous in traffic on any horse. I keep my horse on a private estate and forfeit an arena so I can have safe, fairly stress-free hacking.”*

Themes within the extrinsic factors category included perceptions of other road users, factors around the road itself (surface, road type, volume of traffic), and the availability of access to off-road routes. These factors were perceived as representing a risk that could overcome the risk mitigators of a well-established human-horse relationship or reliable horse behaviour. For example, the following respondents describe that their horses are reliable, but the extrinsic risks presented by the traffic and road conditions still made road use too dangerous to contemplate:

*“we have a very busy road beside us which I wouldn’t use as it has no verge, is poorly lit and drivers speed and drive erratically anyway I wouldn’t take my horse on it despite her being virtually bombproof”*

*“I avoid public roads at all costs due to the traffic. Although my horse is quite road savvy, I don’t think the risk of riding on a main road is worth it”*

The most frequently mentioned extrinsic factor which led to decisions to no longer use roads was dangerous driving (described as people driving too fast, not passing with enough space between the vehicle and horse, and lack of “respect” for horses and riders/handlers, as well as a lack of understanding for horses). Road users were also often described as careless and reckless, lacking humanity:

*“A car driving too fast ignored my request to slow down resulting in my horse panicking and throwing us into a hedge. I fell off and was dragged into the road. Car stopped only so they didn’t hit me then drove off without checking I was OK”*

However, while the most frequently mentioned risk related to speeding car drivers and motorbikes, it was not only motorised vehicles that were purported to cause potentially dangerous situations for horses; a number of respondents mentioned cyclists:

*“The main reason I don’t use the roads now is the bikes. You can’t hear them they fly past with no warning and have no regard for other road users.”*

A combination of intrinsic and extrinsic factors, therefore, came together to influence equestrians’ individual perceptions of road use for each individual human-horse dyad and each individual locale. 

One factor that could significantly influence the perception of either intrinsic or extrinsic risk was a past experience of an accident or a near-miss. Often, one incident (either the participants’ own experience, or one they had heard about) led to an increased perception of risk:

*“After a livery had a serious accident with her horse and both lucky to be alive I won’t let my son hack out on public roads now.”*

*“Years ago I sadly witnessed a runaway horse killed by a car, it made me nervous of riding on the road.”*

As such, roads were perceived as highly dangerous places, where horses and their riders or handlers were extremely vulnerable, and as such presented too high a risk to be considered a viable option, as described by one respondent:

*“I value and love my horse too much to see him hit by a car would break my heart* 🖤*”*

### 3.6. Horse-Related Road Incident Experiences and Views around Incident Reporting

A summary of equestrians’ horse-related road incident experiences is detailed in Table 4. Most participants reported having experienced a horse-related road incident in the previous year. These included incidents that made them or their horse feel unsafe (71.3%), such as verbal abuse or excessive noise from revving engines or vehicle drivers sounding their horn, and incidents that had the potential to cause injury to them or their horse (67.7%). Incidents resulting in injury to either the rider/handler or their horse were reported by 6.1% of participants. When asked how they felt about using roads with their horses, most said they felt slightly anxious (41.4%), 28.7% felt moderately anxious, and 16.4% felt extremely anxious. Only 9.8% said they did not feel at all anxious. There was a relationship between participants’ age category and their level of anxiety when using roads with their horses (*p* < 0.001, *χ*^2^ 70.5). Feeling not at all anxious when using roads was more frequently reported by equestrians younger than 25 years, while feeling extremely anxious when using roads was more frequently reported by equestrians older than 45 years.

Most participants were aware that they were able to report horse-related incidents to the BHS Horse Incidents website and the median score, out of 10, for how likely they were to report an incident was 7 (interquartile range 5 to 10). However, 6.5% (*n* = 337/5169) said they were not at all likely to report an incident. When asked to explain in their own words what would make them more or less likely to report an incident, respondents frequently described feeling that their reporting would not “make a difference” by resulting in change. Participants described wanting changes that were related to their specific case (for example, a driver is reported to the police or fined), or to be able to help reduce the incidence of road-related incidents overall, for other riders.

#### 3.6.1. Factors Associated with Having Had a Near-Miss in the Previous Year

Ten factors were found to be associated with having had an incident in the previous year which had the potential to cause injury to the equestrian or their horse (Table 5). Having had a near-miss in the previous year was associated with: (i) Region of the UK with higher odds of reported near-misses in the North West (OR 2.0, CI 1.4, 2.8), Yorkshire and The Humber (OR 1.8, CI 1.2, 2.6), West Midlands (OR 1.4, CI 1.0, 1.9) and South West (OR 1.3, CI 1.0, 1.7) compared to Scotland; (ii) the frequency of weekly road use with odds increasing as the frequency of road use increased (*p* < 0.001); (iii) the proximity of local off-road routes with odds increasing with increasing distance to the nearest off-road route (*p* < 0.001); (iv) how well connected the local off-road network is with odds increasing as off-road routes were less well connected (*p* < 0.001); (v) the level of anxiety when using roads with their horses with odds increasing as the level of anxiety increased (*p* < 0.001); (vi) years of equestrian experience with odds increasing as the years of experience increased (*p* = 0.002); (vii) equestrians’ age with odds increasing as age decreased (*p* < 0.001); and the type of exercise they did with their horses on the road with odds higher when horses were (viii) ridden (OR 1.9, CI 1.5, 2.5), (ix) driven in a carriage (OR 1.7, CI 1.2, 2.5) or (x) ridden while leading another ridden horse (OR 1.6, CI 1.1, 2.2). Univariable results of the near-miss incidents model are presented in Table S3.

The Hosmer–Lemeshow goodness-of-fit test (*p* = 0.20, *χ*^2^ 6.04) indicated the logistic regression model described the data well. The area under the ROC curve was 0.74 (CI 0.72, 0.75), suggesting the model had sufficient overall predictive power.

#### 3.6.2. Factors Associated with Having Had an Injury-Causing Incident in the Previous Year

Following multivariable analysis, we identified four factors associated with an equestrian having had an injury-causing incident while using the roads with their horse in the previous year; this included injury to the equestrian and/or the horse (Table 6). Having experienced an injury-causing incident in the previous year was associated with: (i) The proximity of local off-road routes, with odds higher when the nearest off-road route was more than 1 mile away (*p* = 0.015); (ii) extreme anxiety when using roads with a horse (OR 2.3, CI 1.3, 4.0) or no longer doing so (OR 2.5, CI 1.1, 5.8); (iii) when the equestrian reported having also had a near-miss in the previous year (OR 6.0, CI 3.8, 9.6); and (iv) riding and leading a ridden horse on the road (OR 2.0, CI 1.4, 3.1). Univariable results of the injury-causing incidents model are presented in Table S4.

The Hosmer–Lemeshow goodness-of-fit test (*p* = 0.76, *χ*^2^ 1.86) indicated the logistic regression model described the data well. The area under the ROC curve was 0.72 (CI 0.69, 0.74), suggesting the model had sufficient overall predictive power.

## 4. Discussion

This paper reports the first UK-wide study describing equestrians’ use of public roads as part of their horse’s exercise regime. Public roads are common places where conflict and injury occur, and therefore represent an interesting and understudied “One Health” problem, in which the wellbeing of horses, their handlers, and other road users become interconnected as the parties try to share the same space. This study provides an understanding of how, why, and when equestrians use roads, as well as the factors that are connected to the occurrence of near-misses and injuries. The data highlight complex interplay between the road itself, the horse-human dyad (for example, their relationship, the horse’s confidence in traffic and the equestrian’s confidence in the horse), and other road users.

Considering that the questionnaire was available for only a month and a half, we received a very good response from horse owners and carers, with over 6000 initial responses, indicating that the topics covered in this study were of high interest to the UK equestrian community. Most equestrians (97%) reported exercising one or more of their horses at least once per week. This exercise frequency is in keeping with a previous study which looked at owner-reported activities and exercise in 797 horses in Great Britain between 2009 and 2011, where 82.8% of horses were reported to have taken part in exercise activities in the previous week [18]. Horses were exercised in a variety of ways, including riding, in-hand or groundwork, carriage driving and/or riding and leading, with exercise times and distances generally greater on weekend days compared to weekdays. The variation in weekday and weekend exercise is likely due to equestrians having more time to spend with their horses outside of the conventional working week and is consistent with reports of more frequent riding and greater distances covered on weekend days by horse riders in Devon [2]. This increased exercise activity on weekends could translate into health and welfare benefits for both the human and their horse. Human health research has shown that physical activity conducted on weekends plays an important role in long-term weight management [19,20,21]. Obtaining objectively-measured exercise times and distances for equestrians would be of benefit to corroborate the self-reported approximations.

A large proportion of equestrians incorporated off-road routes (77%) and roads (84%) at least once per week into their exercise sessions, with approximately 9% reporting that they almost exclusively used roads to exercise their horses. The sampled population of equestrians, therefore, represents a large proportion of regular ‘hackers’ in contrast to the population sampled by Wylie et al. [18], where only 50.7% of horses had undertaken ridden hacking activity in the previous week. However, our definition of hacking (using a combination of roads and off-road routes to exercise your horse) included both ridden and non-ridden activities and may account for some of the increase in the proportion of equestrians that hacked. There were variations in the reported availability, proximity and connectivity of the local off-road networks available to equestrians. More than half of the equestrians felt that there were few off-road options local to where their horses were kept and that off-road routes, where available, were often separated by considerable amounts of road. While approximately half of the equestrians said that the nearest off-road route was within one mile, just over 8% reported their nearest off-road routes were more than five miles away. We anticipated that, similar to walkers and cyclists [22,23], the proximity and connectivity of safe, off-road riding spaces would have an impact on equestrian activity and road use. Evidence of this was indeed discovered in our subsequent analyses.

We also identified regional differences in off-road route availability with equestrians from Northern Ireland, Wales, the West Midlands and the North West reporting lower accessibility to, and poorer connectivity of, off-road routes in contrast to equestrians from Scotland. This is likely because legislation around public off-road rights of way and access to the countryside is different in Scotland compared to the rest of the UK. In England and Wales, ridden and led horses are restricted to using rights of way designated as bridleways (including pedestrian and cyclists), restricted byways (including bridleway user groups and horse-drawn vehicles) and byways open to all traffic [24]. In Northern Ireland, access legislation is, again, slightly different with seemingly fewer provisions made for equestrians [25]. The Land Reform (Scotland) Act 2003 introduced a right of responsible access to most of the Scottish countryside in 2005, allowing non-motorised access to everyone (including ridden and led horses and horse-drawn vehicles) across land and inland water in Scotland as long as they behave responsibly and follow the Scottish Outdoor Access Code [26]. Some exclusions apply; for example, agricultural land where crops are growing; however, horses can be exercised on crop field margins. This means that equestrians in Scotland are not restricted to designated off-road routes as in the rest of the UK, but can use most existing off-road routes if it is safe and responsible for them to do so. However, it is important to keep in mind that even though off-road routes may exist, it does not necessarily mean that they are well-maintained or accessible.

It is well-established that exercising in green spaces (“green exercise”) has positive impacts on both physical and mental health [27,28,29]. A study of 263 people taking part in various green exercise activities (including walking, cycling and horse riding) in the UK found significant improvements in self-esteem and negative feelings, such as tension, anger and depression, post-exercise compared to pre-exercise [27]. These improvements in self-esteem and mood were independent of the type, duration or intensity of the green exercise. Additionally, people exercising outdoors or in green-coloured environments report lower levels of perceived exertion even though the physiological exertion is greater, suggesting outdoor exercise is perceived to be easier, and therefore, more likely to be maintained [30,31]. Despite the evident benefits of equestrian activities on human health and wellbeing [32,33], equestrians are often excluded from policy and planning that pertains to recreational use and access to green spaces. The proximity of green spaces, the number and type of roads that require crossing, the lack of safe pedestrian or cycling spaces and the volume and/or speed of traffic have all been identified as barriers to physical activity by people walking and cycling [9,12,27]. It is highly likely that similar barriers affect equestrians and contribute to their decisions to use or avoid roads and ultimately impact their physical and psychological health, as well as that of their horses.

Therefore, we wanted to investigate what impacts equestrian road use, using both quantitative and qualitative methods. The quantitative analysis identified regional differences in road use which reflected the regional differences in availability and connectivity of off-road networks. Equestrians in the North West, Wales and West Midlands were more likely to use roads compared to equestrians in Scotland. Interestingly, equestrians in Northern Ireland, while reporting the least off-road access options, were not more likely to use roads, suggesting that if the barriers are too great, equestrians may limit their exercise sessions; this was reflected by equestrians in Northern Ireland also tending to cover shorter daily distances with their horses. The South West and Yorkshire also had higher odds of equestrian road use, suggesting that local areas within regions may also differ but, perhaps also that better off-road options will encourage equestrians to go further, and therefore, result in some road use. Indeed, road use was associated with equestrians covering greater distances with their horses on weekend days, indicating that avoiding roads may have a direct impact on the length of exercise sessions. As expected, road use was influenced by the proximity and connectivity of off-road routes; equestrians were most likely to use roads when the nearest off-road route was within one to two miles away compared to more than five miles away and when off-road routes were less well connected, although not in an entirely linear fashion. A similar relationship was found between active modes of travel and the proximity of shops and cycling routes in urban areas; people were more likely to walk or cycle when cycling facilities and retail were closer, but the relationship was not entirely linear [22]. Odds of road use were highest when off-road routes were well or poorly connected, but lower when off-road routes were very poorly connected.

Road use was also associated with equestrians’ age groups, with road use more likely in younger age categories. This may reflect that younger equestrians are less risk-averse and feel more confident to use roads with their horses. It is well established that young novice drivers tend to underestimate accident risk, overestimate their own driving skills and are more willing to accept risk when driving compared to more experienced drivers [34]. Indeed, we found that younger equestrians were less likely to feel anxious about using roads compared to older equestrians. Previous equestrian research has identified that younger riders are also more likely to be involved in road collisions [2,3] which may in part reflect the fact that they are also more likely to use roads. A study of perceived collision or incident risk in equestrians in Norway found that negative attitudes towards other road users increased with increasing equestrians’ age, suggesting that greater exposure to potentially risky situations in traffic lead to more negative attitudes, which could contribute to older equestrians being less likely to use roads [4].

Finally, road use also depended on the type of exercise equestrians undertook with their horses. Odds of road use were higher in leisure riding and carriage driving equestrians, but lower in equestrians who either exercised their horses in-hand or via ridden schooling or training. This seems logical because in-hand work and ridden schooling often take place in designated grass or surfaced training areas, but the direction in which this association flows is unclear; are equestrians doing these types of activities because they are avoiding road use or are they not using roads because they are doing these types of activities? It also serves to highlight that ridden and carriage driven horses are more likely to use roads, and are, therefore, both vulnerable equestrian road user groups, yet most of the equestrian road safety messaging and images focus on the ridden horse. Interestingly, equestrians who took part in competitive activities were not significantly more or less likely to use roads, indicating a lack of clear distinction between leisure and competitive riders.

Qualitative content analysis of comments from equestrians who no longer use roads with their horses revealed a complex interplay between extrinsic (road and traffic-related) and intrinsic (horse/handler related) factors overshadowed by strong feelings of danger, compromised safety and conflict with other road users. Equestrians who had access to primarily off-road routes felt ‘lucky’ to not have to use roads with some even relaying how they had moved their horses to a yard with better off-road options, even if they had to travel considerably further to care for their horses, just so they could avoid using roads. A useful comparison can be drawn between the results of this study and the results of studies detailing the experience of another set of vulnerable road users who have received more research attention than equestrians—cyclists. Like the equestrian respondents in this study, cyclists are also reported to feel extreme anxiety about being in traffic, preferring off-road routes when possible [35,36]. Both equestrians and cyclists position themselves as exposed, vulnerable road users who are at the mercy of other road users; those other road users are often thought to lack understanding of the needs of more vulnerable road users. Much effort has, therefore, been spent on providing off-road cycle networks [37,38] and educating drivers about the needs of cyclists [39].

However, for equestrian road users there have been fewer available funds for off-road networks, and campaigns for driver awareness of equestrian needs appear to have more limited uptake. Moreover, this study showed that equestrians also experienced conflict with cyclists, which is problematic because cyclists and equestrians are also expected to share space on off-road routes. It is, therefore, imperative that vulnerable road user groups work together, not only to campaign for safer roads, but also to share information with their users about safe on-road and off-road interactions with one another. For example, the BHS and Cycling UK came together to develop the “Be Nice, Say Hi” campaign, which aims to promote positive social interactions between cyclists and equestrians on shared routes and ultimately improve safety for both groups when interacting with each other [40].

Equestrians in this study felt strongly that other road users were unaware of the nature of horses, and furthermore felt that roads were so dangerous that they could not habituate nervous or young horses to roads without creating a safety hazard. Importantly we have shown that road use is not just limited to those *riding* their horses, but also to people carriage driving, leading, long-reining, or riding and leading another horse; each activity represents different levels of risk. The results, therefore, suggest that campaigns which teach other road users about horse behaviour could be beneficial in encouraging other road users to give horses more room, and pass more slowly. Most campaigns, thus, far have employed traditional “warning” wording about the dangers of passing horses too quickly. However, studies of behaviour change in road use have highlighted the better response gained from positively framed and inclusive messaging, which makes an example of the desired behaviours [41] (as in the “Be Nice, Say Hi” campaign), rather than traditional warning messages which focus on riding specifically.

As many equestrians had expressed safety concerns and danger of injury to them or their horse when using roads, next we wanted to quantitatively explore what factors were associated with having had a near-miss or injury-causing incident in the previous year. Near-misses were associated with the region, frequency of weekly road use and proximity and connectivity of off-road routes. Regions previously identified as having higher equestrian road use were also associated with a higher risk of near-misses. Similarly, the further away the nearest off-road route was and the less well-connected the off-road network, the more likely it was for a near-miss to have occurred. Additionally, increasing frequency of weekly road use was strongly associated with increased odds of having had a near-miss. Although it is logical to assume that those equestrians who use roads more are also more likely to experience a near-miss, this study is the first to show strong evidence of this effect and to quantify the risk. In addition, we show that lack of proximity to off-road options, which are also well connected, plays an important role in experiences of near-misses, meaning that increasing time spent on roads leads to higher risk. There was an inverse relationship between odds of having had a near-miss and the age of the rider/handler, with younger riders having higher odds. Similar age-related differences in near-miss experiences have been found in cyclists. Cyclists older than 55 years were found to have lower rates of near-misses compared to younger cyclists in a study of 1532 diary days documenting cycling trips and incidents, which was attributed to their greater experience and a higher likelihood of cycling for recreation rather than commuting [42]. However, contrary to expectation, we found that increasing years of equestrian experience were associated with higher odds of having had a near-miss. This is in contrast to findings by the UK Near Miss project, where new cyclists had higher near-miss rates compared to more experienced cyclists and were also more likely to perceive incidents as being a near-miss, or as deliberate or scary [43]. This is likely because the relationship between age and equine experience is not linear; some young riders that have grown up around horses may have more years of equine experience compared to older equestrians who took up riding later in life. Our findings suggest that unlike cyclists, who may appear to become habituated to near-misses over time and are, therefore, less likely to report a risky experience as being a near-miss [43], equestrians, and their horses, may become less tolerant of risky situations as their exposure to road use increases. Having had a near-miss in the previous year was strongly associated with increasing levels of anxiety regarding road use. Although it is not possible to definitively determine the direction of this association, whether the near-miss contributed to the anxiety or vice versa, it is interesting to note that near-misses were associated with a linear increase in anxiety, as well as with ceasing to use roads altogether. The perceived risk of having a road-related incident or collision increased as the number of past near-misses experienced by equestrians in Norway increased [4]. This suggests that near-miss experiences do contribute to road avoidance and potentially perpetuate any existing road use anxiety. Finally, near-misses were more common in equestrians who exercised their horses on roads by riding, carriage driving and riding while leading a ridden horse. The latter is particularly interesting as most equestrians would ride their horse and lead a child rider on a pony, indicating that other road users failed to behave appropriately, even around children. It is potentially also more difficult to control two horses when things go wrong compared to when one person is on foot, which can contribute to dangerous interactions with other road users. Collectively, these findings strongly suggest that it is not the occasional, inexperienced equestrian road user that is more likely to experience a dangerous near-miss situation but, rather, near-misses are more dependent on the frequency of road use, time spent on roads, availability of alternative off-road options and that younger, but not necessarily less experienced, equestrians were at highest risk.

There were significant similarities between the near-miss and injury models in terms of proximity to nearest off-road routes, anxiety around using roads and in what capacity equestrians used roads. Additionally, having had a near-miss in the previous year was associated with also having had an incident that resulted in injury to either the equestrian or their horse. Although it is not possible to tell which occurred first, the near-miss or the injury, it does present a potential risk escalation scenario with those experiencing near-misses then much more likely to go on to be involved in an injury-causing incident. Interestingly, most road safety casualty measures are based on injury-causing incidents that focus on severe and fatal injuries [44,45]. While these are undoubtedly of importance, near-misses and slight injuries are much more frequent, but are often overlooked as they are not considered to be accurately represented [46] or are not perceived to lead to serious consequences. However, in terms of injury prevention for vulnerable road users, we feel that more emphasis should be placed on collating near-miss and slight injury data and communicating with road users that cause these incidents because if we can intervene at this stage, we are much more likely to change behaviour which may otherwise escalate to cause serious injury or even death. Equestrians who had an injury-causing incident were also much more likely to be extremely anxious about using roads and also to have ceased using roads with their horses, suggesting a direct trauma from the incident itself. Once again, riders who rode while leading a ridden horse on the road were also more likely to have had an injury-causing incident in the previous year. As discussed, children are often led on ponies by their parents, and it is extremely concerning that this group of equestrians is more likely to be injured.

Comparisons with cycling help to contextualise some of the results found in this study—but, of course, there is one missing element from cycle research that is relevant to the present study: a living, breathing half-tonne animal who may have its own fears and anxieties over road use [8,47,48]. One particularly interesting finding in this study was that higher levels of human anxiety were related to a higher risk of having had a near-miss or injury-causing incident in the past year, although the direction of this association requires further investigation. It is known that rider/handler anxiety can transmit to the horse [49], who may then become more fearful–potentially increasing the risk of behaviour, such as spooking or bolting. This could lead to an accident for the rider or other road users. The findings in this study suggest that, apart from addressing the road environment and behaviour of other road users, support for anxious riders and building confidence in themselves and their horses, could assist in helping them cope better on the roads and contribute to improved road safety.

### Limitations

The exercise times and distances reported here are approximations, and it is likely that some equestrians either over- or under-estimate the times and/or distances reported. Additionally, the availability of off-road riding routes was based on equestrians’ perception rather than an accurate mapping of equestrian off-road routes in each area. Lastly, as this was a cross-sectional study conducted in the UK in the autumn of 2020, equestrians were asked to report in general on their average activities, and seasonal variation in road use or exercise frequency was not taken into account. For example, equestrians reporting on their riding habits in Devon report riding less often and for shorter durations in the winter months compared to the summer [2]. Additionally, different regions of the UK were under varied tiers of lockdown, due to the COVID-19 pandemic at the time the questionnaire was filled out, which may have affected equestrian activities.

## 5. Conclusions

This study represents the first UK-wide description of equestrian road use in the UK, and has highlighted the frequency of injuries and near-misses, as well as the extreme anxiety felt by equestrians on the road. This previously under-researched area represents an important field of study for both human and horse wellbeing, given the high risk of physical or psychological harm, and sometimes death. Horse riders and handlers preferred to keep off roads when possible, but often found it impossible to exercise their horses without some road use. As a result of not having access to off-road routes, some equestrians simply stopped exercising their horses at all–a response which could have potential equine welfare implications, such as weight gain, obesity and secondary health consequences, such as laminitis [50,51,52].

These results, therefore, support the need for targeted campaigns around encouraging responsible behaviour of other road users around horses, ideally explaining how road users should behave around horses and the reasons behind this behaviour. Further, this project has highlighted the need for increased off-road options for equestrians, who are often forgotten or ignored in the development of “green exercise” initiatives. The importance of safe riding areas is particularly relevant given the finding in this study that children and young people are at increased risk of having road-related incidents.

## Figures and Tables

**Table 1:** A summary of the demographics of UK equestrians (*n* = 5817) that completed a questionnaire regarding their activity when caring for and exercising their horses, ponies, donkeys or mules.

Variable	Number of Participants	Percentage of Participants	95% Confidence Interval (%)
**Country (*n* = 5817)**			
England	4488	77.2	76.1, 78.2
Wales	674	11.6	10.8, 12.4
Scotland	590	10.1	9.4, 10.9
Northern Ireland	65	1.1	0.8, 1.4
**Region (*n* = 5212)**			
East of England	743	14.3	13.3, 15.2
East Midlands	419	8.0	7.3, 8.8
Greater London	20	0.4	0.2, 0.6
North East	117	2.2	1.8, 2.6
North West	418	8.0	7.3, 8.8
Northern Ireland	65	1.2	0.9, 1.5
Scotland	590	11.3	10.5, 12.2
South East	721	13.8	12.9, 14.8
South West	806	15.5	14.5, 16.4
Wales	674	12.9	12.0, 13.8
West Midlands	375	7.2	6.5, 7.9
Yorkshire and The Humber	264	5.1	4.5, 5.7
**Equestrians’ age category (*n* = 5006)**			
Under 18 years	110	2.2	1.8, 2.6
18–24 years	335	6.7	6.0, 7.4
25–34 years	533	10.7	9.8, 11.5
35–44 years	864	17.3	16.2, 18.3
45–54 years	1294	25.9	24.6, 27.1
55–64 years	1336	26.7	25.5, 27.9
65–74 years	507	10.1	9.3, 11.0
>74 years	27	0.5	0.3, 0.7
**Equestrian experience (*n* = 5011)**			
<1 year	13	0.3	0.1, 0.4
1–5 years	177	3.5	3.0, 4.0
6–10 years	286	5.7	5.1, 6.3
11–15 years	382	7.6	6.9, 8.4
16–20 years	515	10.3	9.4, 11.1
21–25 years	231	4.6	4.0, 5.2
26–30 years	513	10.2	9.4, 11.1
>30 years	2894	57.8	56.4, 59.1
**British Horse Society membership (*n* = 5011)**			
No	2334	46.6	45.2, 48.0
Yes	2677	53.4	52.0, 54.8
**Number of horses owned/cared for (*n* = 5817)**			
1	2359	40.6	39.3, 41.8
2	1717	29.5	28.3, 30.7
3	844	14.5	13.6, 15.4
4	388	6.7	6.0, 7.3
5	202	3.5	3.0, 3.9
6	103	1.8	1.4, 2.1
>6	204	3.5	3.0, 4.0
**Where horses are kept (*n* = 5817)**			
Home	1810	31.1	29.9, 32.3
“Do-it-yourself” livery	1795	30.9	29.7, 32.0
Rented private yard/field	930	16.0	15.0, 16.9
Assisted livery	453	7.8	7.1, 8.5
Full livery	370	6.4	5.7, 7.0
Working livery	99	1.7	1.4, 2.0
Other	360	6.2	5.6, 6.8

**Table 2:** A summary of UK equestrians’ (*n* = 5335) activity around using off-road routes and roads to exercise their horses, ponies, donkeys and mules.

Variable	Number of Participants	Percentage of Participants	95% Confidence Interval (%)
**Frequency of use of off-road routes (*n* = 5334)**			
Don’t use them	472	8.9	8.1, 9.6
Infrequently (<1/week)	744	14.0	13.0, 14.9
1–2 days/week	1325	24.8	23.7, 26.0
2–3 days/week	1022	19.2	18.1, 20.2
3–4 days/week	770	14.4	13.5, 15.4
4–5 days/week	466	8.7	8.0, 9.5
5–6 days/week	262	4.9	4.3, 5.5
6–7 days/week	273	5.1	4.5, 5.7
**Frequency of use of tolled off-road routes (*n* = 5334)**			
Don’t use them	4720	88.5	87.6, 89.3
Infrequently (<1/week)	295	5.5	4.9, 6.1
1–2 days/week	148	2.8	2.3, 3.2
2–3 days/week	71	1.3	1.0, 1.6
3–4 days/week	46	0.9	0.6, 1.1
4–5 days/week	39	0.7	0.5, 1.0
5–6 days/week	9	0.2	0.1, 0.3
6–7 days/week	6	0.1	0.0, 0.2
**Availability of local off-road routes (*n* = 5256)**			
Very few	1773	33.7	32.5, 35.0
Few	1021	19.4	18.4, 20.5
Moderate	927	17.6	16.6, 18.7
Several	826	15.7	14.7, 16.7
Spoilt for choice	682	13.0	12.1, 13.9
Don’t know	27	0.5	0.3, 0.7
**Distance to nearest off-road route (*n* = 5256)**			
Within 1 mile	2676	50.9	49.6, 52.3
Between 1–2 miles	1338	25.5	24.3, 26.6
Between 2–5 miles	701	13.3	12.4, 14.3
>5 miles	432	8.2	7.5, 9.0
Don’t know	109	2.1	1.7, 2.5
**How well connected the local off-road network is (*n* = 5256)**			
Very poorly connected	1813	34.5	33.2, 35.8
Poorly connected	1301	24.8	23.6, 25.9
Adequately connected	1069	20.3	19.3, 21.4
Well connected	553	10.5	9.7, 11.4
Very well connected	380	7.2	6.5, 7.9
Don’t know	140	2.7	2.2, 3.1
**Use roads to exercise their horses (*n* = 5426)**			
No	346	6.4	5.7, 7.0
Yes	5080	93.6	93.0, 94.3
**Frequency of road use (*n* = 5335)**			
Don’t use them/infrequently (<once/week)	854	16.0	15.0, 17.0
1–2 days/week	1226	23.0	21.9, 24.1
2–3 days/week	1058	19.8	18.8, 20.9
3–4 days/week	844	15.8	14.8, 16.8
4–5 days/week	574	10.8	9.9, 11.6
5–6 days/week	308	5.8	5.1, 6.4
6–7 days/week	471	8.8	8.1, 9.6
**Proportion of road to off-road exercise (*n* = 5172)**			
100% road	472	9.1	8.3, 9.9
80% road, 20% off-road	1485	28.7	27.5, 29.9
50% road, 50% off-road	1233	23.8	22.7, 25.0
20% road, 80% off-road	1476	28.5	27.3, 29.8
100% off-road	506	9.8	9.0, 10.6

**Table 3:** Multivariable logistic regression modelling to identify factors associated with road use by UK’s equestrians (based on responses from 4842 equestrians in October 2020).

Variable	Coefficient	Standard Error	Odds ratio (OR)	95% Confidence Interval (OR)	Wald *p*-Value; LRS *p*-Value *
**Region of the UK**					<0.001 *
Scotland	Reference				
East of England	0.19	0.22	1.2	0.8, 1.8	0.385
East Midlands	0.42	0.27	1.5	0.9, 2.6	0.121
South East and London	0.20	0.22	1.2	0.8, 1.9	0.347
North East	1.02	0.55	2.8	0.9, 8.2	0.064
North West	1.22	0.31	3.4	1.8, 6.2	<0.001
Northern Ireland	0.31	0.51	1.4	0.5, 3.7	0.543
South West	1.47	0.28	4.4	2.5, 7.5	<0.001
Wales	0.92	0.27	2.5	1.5, 4.3	0.001
West Midlands	0.60	0.28	1.8	1.0, 3.1	0.033
Yorkshire and the Humber	0.70	0.33	2.0	1.1, 3.9	0.033
**Approximate daily exercise distance during the weekend**					<0.001 *
Up to 1 mile	Reference				
1–2 miles	0.38	0.24	1.5	0.9, 2.3	0.120
2–5 miles	1.60	0.23	5.0	3.2, 7.8	<0.001
5–7 miles	1.96	0.26	7.1	4.3, 11.7	<0.001
7–10 miles	2.01	0.30	7.4	4.1, 13.5	<0.001
10–12 miles	2.21	0.42	9.1	4.0, 20.7	<0.001
12–15 miles	2.11	0.56	8.3	2.8, 24.6	<0.001
>15 miles	1.28	0.40	3.6	1.6, 7.9	0.001
Not sure	0.49	0.27	1.6	1.0, 2.8	0.066
**Distance to nearest off-road route**					0.004 *
Within 1 mile	0.41	0.24	1.5	0.9, 2.4	0.088
Between 1–2 miles	0.88	0.26	2.4	1.4, 4.0	0.001
Between 2–5 miles	0.19	0.26	1.2	0.7, 2.0	0.453
>5 miles	Reference				
Don’t know	−0.05	0.42	1.0	0.4, 2.2	0.910
**Flow of local off-road riding routes**					<0.001 *
Very poorly connected	1.19	0.22	3.3	2.1, 5.0	<0.001
Poorly connected	1.56	0.22	4.7	3.1, 7.3	<0.001
Adequately connected	1.38	0.22	4.0	2.6, 6.1	<0.001
Well connected	1.55	0.27	4.7	2.7, 8.1	<0.001
Very well connected	Reference				
Don’t know	1.23	0.39	3.4	1.6, 7.4	0.002
**Equestrians’ age category**					<0.001 *
Under 18 years	1.18	0.53	3.2	1.1, 9.1	0.027
18–24 years	1.06	0.32	2.9	1.5, 5.4	0.001
25–34 years	0.75	0.29	2.1	1.2, 3.7	0.009
35–44 years	0.35	0.23	1.4	0.9, 2.2	0.137
45–54 years	0.49	0.22	1.6	1.1, 2.5	0.028
55–64 years	−0.01	0.21	1.0	0.7, 1.5	0.957
>64 years	Reference				
**Horses exercised by leisure riding**					
No	Reference				
Yes	1.45	0.17	4.3	3.0, 6.0	<0.001
**Horses exercised by carriage driving**					
No	Reference				
Yes	1.08	0.38	2.9	1.4, 6.1	0.004
**Horses exercised in-hand**					
No	Reference				
Yes	−0.25	0.13	0.8	0.6, 1.0	0.049
**Horses exercised by ridden schooling/training**					
No	Reference				
Yes	−0.42	0.15	0.7	0.5, 0.9	0.004

^*^ LRS—Likelihood ratio statistic.

**Table 4:** A summary of UK equestrians’ (*n* = 5171) horse-related road incident experiences.

Variable	Number of Participants	Percentage of Participants	95% Confidence Interval (%)
**How do you feel about using roads with your horse? (*n* = 5171)**			
Not at all anxious	509	9.8	9.0, 107
Slightly anxious	2143	41.4	40.1, 42.8
Moderately anxious	1484	28.7	27.5, 29.9
Extremely anxious	850	16.7	15.4, 17.4
Don’t use roads	185	3.6	3.1, 4.1
**Have you had an incident on the road in the previous year that made you or your horse feel unsafe? E.g., verbal abuse or excessive noise (*n* = 5122)**			
No	1469	28.7	27.4, 29.9
Yes	3653	71.3	70.1, 72.6
**Have you had an incident on the road in the previous year which had the potential to cause injury to you or your horse? (*n* = 5122)**			
No	1657	32.4	31.1, 33.6
Yes	3465	67.7	66.4, 68.9
**Have you had an incident on the road in the previous year which resulted in injury to you or your horse? (*n* = 5122)**			
No	4812	94.0	93.3, 94.6
Yes	310	6.1	5.4, 6.7
**Are you aware that you can report horse-related road incidents to the British Horse Society? (*n* = 5171)**			
No	1531	29.6	28.4, 30.9
Yes	3640	70.4	69.1, 71.6

**Table 5:** Multivariable logistic regression modelling to identify factors associated with higher odds of having had a road-related near-miss while using roads with a horse in the previous year (based on responses from 4842 UK equestrians in October 2020).

Variable	Coefficient	Standard Error	Odds Ratio (OR)	95% Confidence Interval (OR)	Wald *p*-Value; LRS *p*-Value *
**Region of the UK**					<0.001 *
Scotland	Reference				
East of England	0.03	0.13	1.0	0.8, 1.3	0.840
East Midlands	0.16	0.15	1.2	0.9, 1.6	0.309
Greater London	−0.50	0.53	0.6	0.2, 1.7	0.343
North East	0.37	0.25	1.5	0.9, 2.4	0.127
North West	0.69	0.16	2.0	1.4, 2.8	<0.001
Northern Ireland	0.26	0.36	1.3	0.6, 2.6	0.470
South East	0.22	0.13	1.2	1.0, 1.6	0.103
South West	0.27	0.13	1.3	1.0, 1.7	0.045
Wales	0.12	0.15	1.1	0.9, 1.5	0.397
West Midlands	0.32	0.16	1.4	1.0, 1.9	0.046
Yorkshire and the Humber	0.58	0.19	1.8	1.2, 2.6	0.002
**Frequency of weekly road use**					<0.001 *
Less than once per week/never	Reference				
1–2 days/week	0.81	0.12	2.2	1.8, 2.8	<0.001
2/3 days/week	1.00	0.12	2.7	2.1, 3.5	<0.001
3–4 days/week	1.16	0.13	3.2	2.5, 4.2	<0.001
4–5 days/week	1.21	0.15	3.4	2.5, 4.5	<0.001
5–6 days/week	1.21	0.17	3.4	2.4, 4.7	<0.001
6–7 days/week	1.26	0.16	3.5	2.6, 4.8	<0.001
**Distance to nearest off-road route**					0.001 *
Within 1 mile	Reference				
Between 1–2 miles	0.29	0.09	1.3	1.1, 1.6	0.001
Between 2–5 miles	0.37	0.12	1.5	1.2, 1.8	0.002
>5 miles	0.38	0.15	1.5	1.1, 1.9	0.011
Don’t know	0.11	0.27	1.1	0.7, 1.9	0.678
**Flow of local off-road riding routes**					<0.001 *
Very poorly connected	0.80	0.14	2.2	1.7, 2.9	<0.001
Poorly connected	0.59	0.14	1.8	1.4, 2.4	<0.001
Adequately connected	0.52	0.14	1.7	1.3, 2.2	<0.001
Well connected	0.37	0.15	1.4	1.1, 2.0	0.015
Very well connected	Reference				
Don’t know	0.39	0.26	1.5	0.9, 2.5	0.142
**Level of anxiety when using roads with their horse**					<0.001 *
Not at all anxious	Reference				
Slightly anxious	0.88	0.11	2.4	1.9, 3.0	<0.001
Moderately anxious	1.50	0.12	4.5	3.5, 5.7	<0.001
Extremely anxious	2.11	0.15	8.2	6.1, 11.1	<0.001
No longer use roads	1.06	0.24	2.9	1.8, 4.6	<0.001
**Equestrian experience**					0.002 *
Up to 5 years	Reference				
6–10 years	0.48	0.21	1.6	1.1, 2.5	0.022
11–15 years	0.50	0.20	1.6	1.1, 2.5	0.014
16–20 years	0.68	0.20	2.0	1.3, 2.9	0.001
21–25 years	0.69	0.23	2.0	1.3, 3.1	0.002
26–30 years	0.72	0.20	2.1	1.4, 3.0	<0.001
>30 years	0.76	0.17	2.1	1.5, 3.0	<0.001
**Equestrians’ age category**					<0.001 *
Under 18 years	0.89	0.26	2.4	1.5, 4.0	0.001
18–24 years	0.94	0.19	2.5	1.8, 3.7	<0.001
25–34 years	0.72	0.16	2.1	1.5, 2.8	<0.001
35–44 years	0.51	0.13	1.7	1.3, 2.2	<0.001
45–54 years	0.45	0.12	1.6	1.2, 2.0	<0.001
55–64 years	0.31	0.12	1.4	1.1, 1.7	0.011
>64 years	Reference				
**Ride on roads**					
No	Reference				
Yes	0.66	0.13	1.9	1.5, 2.5	<0.001
**Carriage drive on roads**					
No	Reference				
Yes	0.56	0.18	1.7	1.2, 2.5	0.002
**Ride while leading a ridden horse on roads**					
No	Reference				
Yes	0.44	0.18	1.6	1.1, 2.2	0.015

^*^ LRS—Likelihood ratio statistic.

**Table 6:** Multivariable logistic regression modelling to identify factors associated with higher odds of having had a road-related injury-causing incident (injury to equestrian and/or horse) while using roads with a horse in the previous year (based on responses from 5122 UK equestrians in October 2020).

Variable	Coefficient	Standard Error	Odds Ratio (OR)	95% Confidence Interval (OR)	Wald *p*-Value; LRS *p*-Value *
**Distance to nearest off-road route**					0.015 *
Within 1 mile	Reference				
Between 1–2 miles	0.42	0.15	1.5	1.1, 2.0	0.004
Between 2–5 miles	0.44	0.17	1.6	1.1, 2.2	0.011
>5 miles	0.40	0.21	1.5	1.0, 2.2	0.053
Don’t know	0.59	0.39	1.8	0.8, 3.9	0.131
**Level of anxiety when using roads with their horse**					<0.001 *
Not at all anxious	Reference				
Slightly anxious	−0.01	0.28	1.0	0.6, 1.7	0.980
Moderately anxious	0.40	0.28	1.5	0.9, 2.6	0.458
Extremely anxious	0.83	0.29	2.3	1.3, 4.0	0.004
No longer use roads	0.92	0.42	2.5	1.1, 5.8	0.030
**Experienced a near-miss in the previous year**					
No	Reference				
Yes	1.8	0.23	6.0	3.8, 9.6	<0.001
**Ride while leading a ridden horse on roads**					
No	Reference				
Yes	0.71	0.21	2.0	1.4, 3.1	0.001

^*^ LRS—Likelihood ratio statistic.

## Data Availability

The data presented in this study are available upon reasonable request from the corresponding author. The data are not publicly available due to being a part of an on-going project.

## Supplementary Materials

The following are available online at https://www.mdpi.com/article/10.3390/ani11041072/s1, Questionnaire S1: Equine Activity Survey, Table S2: Univariable logistic regression modelling to identify factors associated with road use by UK’s equestrians, Table S3: Univariable logistic regression modelling to identify factors associated with higher odds of having had a road-related near-miss while using roads with a horse in the previous year, Table S4: Univariable logistic regression modelling to identify factors associated with higher odds of having had a road-related injury-causing incident (injury to equestrian and/or horse) while using roads with a horse in the previous year.
