# Supplementary material for: Public Roads as Places of Interspecies Conflict: A Study of Horse-Human Interactions on UK Roads and Impacts on Equine Exercise
Source: Animals (Basel). 2021 Apr 9;11(4):1072. doi: 10.3390/ani11041072 (PMC8070417; doi:10.3390/ani11041072)
Supplement: Supplementary file 1 [file animals-11-01072-s001.pdf]

Article

Public Roads as Places of Interspecies Conflict: A Study of Horse-Human Interactions on UK Roads and Impacts on Equine Exercise

Danica Pollard [1,*] and Tamzin Furtado [2]

[1] Safety Department, The British Horse Society, Abbey Park, Stareton, Kenilworth, Warwickshire CV8 2XZ, UK
[2] Institute of Infection, Veterinary and Ecological Sciences, University of Liverpool, Leahurst Campus, Neston CH64 7TE, UK; tfurtado@liverpool.ac.uk
* Correspondence: dee.pollard@bhs.org.uk; Tel.: +44-2476-840516

Questionnaire S1: Equine Activity Survey

Equine Activity Survey

We would like to know about your activity when caring for and exercising your horse, pony, donkey or mule (all referred to as **horses** from here), and particularly your activity when using public roads and off-road tracks together.

The Department for Transport in the UK publishes annual statistics about the activity of motorists, cyclists and walkers. No such information is currently available for horse owners, loaners or sharers and we would like to change this. We are also interested in seeing to what extent you and your horses use public roads and other rights of way, and your experiences; particularly when using roads. It will be extremely helpful to establish some baseline figures, which will help us with campaigning for improved equestrian road safety and access to safe off-road riding routes.

Currently, we are only collecting information from horse owners/sharers/loaners in England, Wales, Scotland and Northern Ireland.

This survey is part of a larger study investigating equestrian road safety. If you have any questions about the study or this survey, please contact dee.pollard@bhs.org.uk

Questions with * must be answered to complete the submission.

This survey will take approximately 10-15 minutes to complete.

Key/index for terminology used through the survey:

- WEEK – 7 days
- WEEK DAY – Monday to Friday
- WEEKEND DAY – Saturday & Sunday
- HORSE/S - Horse, pony, donkey or mule

Your privacy is important to us. We will only use the information you provide for the purpose of carrying out statistical analysis to support equine safety campaigns and activities. We will never share your data without your explicit consent or unless there is a legal imperative for us to do so. For full details about how we use personal data and to find out more about how we protect your rights, please visit bhs.org.uk/privacy.

FIRST PAGE

1. Which country do you live in? * *Select one option.*

- England
- Scotland
- Wales
- Northern Ireland
- Other

If Other, end of questionnaire

2. Do you currently own, loan or share one or multiple horses, ponies, donkeys or mules (all referred to as **horses** from here)**?***

- Yes
- No

If No, end of questionnaire.

Thank you for your interest but we are currently only collecting information about horse owners/sharers/loaners in England, Wales, Scotland and Northern Ireland.

SECOND PAGE

1. About your activity when travelling to and looking after your horse/s

1.1. How many horses do you currently own/loan/share?* *Please choose a number.*

- 1
- 2
- 3
- 4
- 5
- 6
- 7
- 8
- 9
- 10+

1.2. Where are your horses kept?* *Select one option.*

- At home
- Livery yard (DIY)
- Livery yard (Assisted)
- Livery yard (Full)
- Livery yard (Working e.g. riding school/college)
- Rented private yard or field
- Other, please specify

1.3. What is the main mode of transport you use to travel to your horse/s?* *Select one option.*

- Car
- Motorcycle/moped
- Push bike
- On foot
- Public transport (e.g. bus or train)
- Other, please specify

1.4. Approximately how long does it take you to travel from your home to your horse/s using the main mode of transport selected above?* *Select one option.*

- 0 to 10 minutes
- 10 to 15 minutes
- 15 to 20 minutes
- 20 to 30 minutes
- 30 to 40 minutes
- 40 to 50 minutes
- 50 to 60 minutes
- More than 60 minutes

1.5. What is the approximate distance between your home and where your horse/s are kept?* *Select one option.*

- Less than 1 mile

- 1 to 2 miles
- 2 to 5 miles
- 5 to 7 miles
- 7 to 10 miles
- 10 to 12 miles
- 12 to 15 miles
- 15 to 20 miles
- 20 to 25 miles
- 25 to 30 miles
- More than 30 miles

1.6. In addition to the main mode of transport, what other modes of transport do you use to travel to your horse/s? * *Select all that apply.*

- Car
- Motorcycle/moped
- Push bike
- On foot
- Public transport (e.g. bus or train)
- Other, please specify

1.7. In an average WEEK, how many visits do you make to your horse/s?* *Please choose a number.*

- 1
- 2
- 3
- 4
- 5
- 6
- 7
- 8
- 9
- 10
- 11
- 12
- 13
- 14+

1.8. On an average DAY, how many visits do you make to your horse/s?* *Please choose a number.*

- 1
- 2
- 3
- 4
- 5
- 6
- 7
- 8
- 9
- 10+

1.9. On an average WEEK DAY, approximately how long do you spend caring for your horse/s in a single visit (excluding time spent exercising them)? * *Select one option.*

- Up to 30 minutes
- 30 minutes to 1 hour
- 1 to 2 hours
- 2 to 3 hours
- 3 to 4 hours
- 4 to 5 hours
- More than 5 hours

1.10. On an average WEEKEND DAY, approximately how long do you spend caring for your horse/s in a single visit (excluding time spent exercising them)? * *Select one option.*

- Up to 30 minutes
- 30 minutes to 1 hour
- 1 to 2 hours
- 2 to 3 hours
- 3 to 4 hours
- 4 to 5 hours
- More than 5 hours

THIRD PAGE

2. About your activity with your horse/s

Please fill this section in to include all horses you own/loan/share.

2.1. Do you regularly exercise any of the horses you own/loan/share? * *By regular exercise we mean any in-hand, driving or riding activity you do with your horse/s at least once a week.*

- Yes
- No

2.1.1. If YES, how many of your horses are regularly exercised? *Please choose a number.*

- 1
- 2
- 3
- 4
- 5
- 6
- 7
- 8
- 9
- 10+

If No, please skip to Q 2.21

FOURTH PAGE

2.2. In an average WEEK what type(s) of exercise activities do you and your horse/s participate in? * *Select all that apply.*

- In-hand walking
- In-hand training or schooling
- Lungeing
- Long-lining/long-reining
- Riding – leisure
- Riding – schooling or training
- Riding - competitive
- Carriage driving – leisure
- Carriage driving – schooling or training
- Carriage driving - competitive
- Riding and leading another non-ridden horse
- Riding and leading another ridden horse
- Leading a ridden horse on foot
- Other, please specify

2.3. In an average WEEK, how often do you exercise your horse/s? * *Select one option.*

- 1 to 2 days a week
- 2 to 3 days a week
- 3 to 4 days a week
- 4 to 5 days a week
- 5 to 6 days a week
- 6 to 7 days a week

2.4. On an average WEEK DAY, how long in total do you spend exercising your horse/s? * *Select one option.*

- Up to 30 minutes
- 30 minutes to 1 hour

- 1 to 2 hours
- 2 to 3 hours
- 3 to 4 hours
- 4 to 5 hours
- More than 5 hours
- We don’t usually exercise during the week

2.5. On an average WEEK DAY, approximately what distance in total do you cover with your horse/s? * *Select one option.*

- Less than 1 mile
- 1 to 2 miles
- 2 to 5 miles
- 5 to 7 miles
- 7 to 10 miles
- 10 to 12 miles
- 12 to 15 miles
- 15 to 20 miles
- 20 to 25 miles
- 25 to 30 miles
- More than 30 miles
- Not sure

2.6. On an average WEEKEND DAY, how long in total do you spend exercising your horse/s? * *Select one option.*

- Up to 30 minutes
- 30 minutes to 1 hour
- 1 to 2 hours
- 2 to 3 hours
- 3 to 4 hours
- 4 to 5 hours
- More than 5 hours
- We don’t usually exercise during the weekend

2.7. On an average WEEKEND DAY, approximately what distance in total do you cover with your horse/s? * *Select one option.*

- Less than 1 mile
- 1 to 2 miles
- 2 to 5 miles
- 5 to 7 miles
- 7 to 10 miles
- 10 to 12 miles
- 12 to 15 miles

- 15 to 20 miles
- 20 to 25 miles
- 25 to 30 miles
- More than 30 miles
- Not sure

FIFTH PAGE

2.8. In an average WEEK, how often do you use or cross public roads with your horse/s? * *Select one option.*

- I don't use or cross public roads with my horse/s
- Infrequently – less than once a week
- 1 to 2 days a week
- 2 to 3 days a week
- 3 to 4 days a week
- 4 to 5 days a week
- 5 to 6 days a week
- 6 to 7 days a week

2.9. In an average WEEK, how often do you use free public or private off-road routes with your horse/s? * *Select one option.*

- I don't use free off-road routes with my horse/s
- Infrequently – less than once a week
- 1 to 2 days a week
- 2 to 3 days a week
- 3 to 4 days a week
- 4 to 5 days a week
- 5 to 6 days a week
- 6 to 7 days a week

2.10. In an average WEEK, how often do you use tolled off-road routes with your horse/s (routes you pay a monthly or annual fee to use)? * *Select one option.*

- I don't use tolled off-road routes with my horse/s
- Infrequently – less than once a week
- 1 to 2 days a week
- 2 to 3 days a week
- 3 to 4 days a week
- 4 to 5 days a week
- 5 to 6 days a week
- 6 to 7 days a week

SIXTH PAGE

2.11. How would you best describe the frequency of local off-road routes available to you and your horse/s? * *Select one option.*

- Very few off-road routes
- Few off-road routes
- Moderate amount of off-road routes
- Several off-road routes
- We are spoilt for choice when it comes to off-road routes!
- Don’t know

2.12. How would you best describe the distance that local off-routes are in relation to where your horse is kept? * *Select one option.*

- Close by (within 1 mile)
- A little distance away (between 1 to 2 miles)
- A fair distance away (between 2 to 5 miles)
- A considerable distance away (more than 5 miles)
- Don’t know

2.13. How would you best describe the flow of local off-road routes available to you and your horse/s? * *Select one option.*

- Very poorly connected (routes are separated by lots of road work)
- Poorly connected
- Adequately connected
- Well connected
- Very well connected (routes are separated by little to no road work)
- Don’t know

SEVENTH PAGE

2.14. Which of these exercise activities do you carry out with your horse/s on public roads? * *Select all that apply.*

- I don’t use public roads with my horse/s
- In-hand walking
- Long-lining/long-reining
- Riding
- Carriage driving
- Riding and leading another non-ridden horse
- Riding and leading another ridden horse
- Leading a ridden horse on foot
- Other, please specify

2.15. Approximately what proportion of road work versus off-road work would you and your horse/s do in an average exercise session? * *Select one option.*

- 100% road work
- 80% road work, 20% off-road work

- 50% road work, 50% off-road work
- 20% road work, 80% off-road work
- 100% off-road work

2.16. How would you best describe the type of public roads you and your horse/s regularly use? **Select all that apply.*

- I don't use public roads with my horse/s
- Main roads passing through a town or city
- Residential roads passing through a town or city
- Link roads between towns or cities
- Main roads passing through a village
- Residential roads passing thorough a village
- Link roads between villages or towns
- Other, please specify

2.17. How do you feel about you and your horse/s using a public road? * *Select one option.*

- I don't use public roads with my horse/s
- I don't feel at all anxious
- I feel slightly anxious
- I feel moderately anxious
- I feel extremely anxious

EIGHTH PAGE

2.18. In the previous year, have you experienced an incident while using a public road that made either you or your horse/s feel unsafe (e.g. verbal abuse or excessive noise)? *

- Yes
- No

2.19. In the previous year, have you experienced an incident while using a public road that had the potential to cause injury to you or your horse/s even though it may not have at the time (a near miss)? *

- Yes
- No

2.20. In the previous year, have you experienced an incident while using a public road that resulted in injury to you or your horse/s (an accident)? *

- Yes
- No

NINTH PAGE

2.21. Are you aware that you can report incidents (including accidents and near misses) to the BHS, including incidents that happen off-road? *

- Yes
- No

2.22. On a scale of 0-10 how likely would you be to report a horse-related incident to the BHS? *

- 0 1 2 3 4 5 6 7 8 9 10

  Not at all likely Very likely

2.23. What do you think would make you MORE likely to report a horse-related incident to the BHS?

- Open ended question - long text box.

2.24. What do you think would make you LESS likely to report a horse-related incident to the BHS?

- Open ended question - long text box.

2.25. If you have previously said that you don't use public roads with your horse/s please tell us why.

- Open ended question - long text box.

TENTH PAGE

3. About little bit about you

3.1. Which county do you live in?

- County:

3.2. Which age category represents your age? * *Select one option.*

- Under 18 years
- 18 to 24 years
- 25 to 34 years
- 35 to 44 years
- 45 to 54 years
- 55 to 64 years
- 65 to 74 years
- 75 to 84 years
- More than 84 years

3.3. How long have you been involved with horses? * *Select one option.*

- Less than 1 year
- 1 to 5 years
- 6 to 10 years
- 11 to 15 years
- 16 to 20 years
- 21 to 25 years
- 26 to 30 years
- More than 30 years

3.4. In an average week, what other activities do you take part in to maintain your fitness? * *Select all that apply.*

- Walking/hiking
- Running/jogging
- Cycling
- Gym or an exercise class
- Swimming
- Organised sport
- Other, please specify

3.5. Are you currently a BHS member? *

- Yes
- No

3.6. Would you be interested in taking part in a research project about equestrian road safety? *

- Yes
- No

If yes, please provide your email address:

Eleventh page

Thank you very much for completing the survey to improve our understanding of equine activity.

This survey is part of a larger study investigating equestrian road safety. If you have any questions about the study or this survey, please contact dee.pollard@bhs.org.uk

You can report a horse-related incident to the BHS here: https://www.horseinicidents.org.uk

More information about the work that the BHS does to improve equestrian safety can be found here: https://www.bhs.org.uk/our-work/safety

Table S2. Univariable logistic regression modeling to identify factors associated with road use by UK's equestrians.

Variable	Coefficient	Standard Error	Odds ratio (OR)	95% Confidence Interval (OR)	Wald *p*-Value
**Country (n = 5426)**					
Scotland	Reference				
England	0.61	0.15	1.8	1.4, 2.5	<0.001
Wales	0.85	0.23	2.3	1.5, 3.7	<0.001
Northern Ireland	–0.08	0.43	0.9	0.4, 2.1	0.848
**Region of the UK (n = 4988)**					
Scotland	Reference				
East of England	0.18	0.19	1.2	0.8, 1.8	0.337
East Midlands	0.46	0.24	1.6	1.0, 2.6	0.053
South East & London	0.19	0.19	1.2	0.8, 1.8	0.326
North East	1.13	0.53	3.1	1.1, 8.8	0.032
North West	0.93	0.28	2.5	1.5, 4.4	0.001
Northern Ireland	–0.08	0.43	0.9	0.4, 2.1	0.848
South West	1.36	0.25	3.9	2.4, 6.4	<0.001
Wales	0.85	0.23	2.3	1.5, 3.7	<0.001
West Midlands	0.50	0.25	1.7	1.0, 2.7	0.048
Yorkshire & the Humber	0.62	0.30	1.9	1.0, 3.3	0.040
**Frequency of weekly exericse (n = 5426)**					
1–2 days/week	Reference				
2–3 days/week	0.12	0.21	1.1	0.7, 1.7	0.580
3–4 days/week	0.36	0.21	1.4	1.0, 2.2	0.084
4–5 days/week	0.16	0.20	1.2	0.8, 1.8	0.421
5–6 days/week	–0.1	0.21	1.0	0.7, 1.5	0.959
6–7 days/week	0.10	0.26	0.1	0.7, 1.9	0.689
**Average time spent exercising on a week day (n = 5425)**					
Up to 30 min	Reference				
30 min–1 h	0.31	0.18	1.4	1.0, 1.9	0.080
1–2 h	0.92	0.19	2.5	1.7, 3.7	<0.001
2–3 h	1.10	0.30	3.0	1.7, 5.3	<0.001
3–4 h	1.30	0.53	3.7	1.3, 10.4	0.014
4–5 h	0.30	0.49	1.4	0.5, 3.5	0.540
>5 h	0.26	0.42	1.3	0.6, 3.0	0.538
**Average exercise distance covered on a week day (n = 5423)**					
Up to 1 mile	Reference				
1–2 miles	0.20	0.19	1.2	0.8, 1.8	0.296
2–5 miles	1.10	0.18	3.0	2.1, 4.3	<0.001
5–7 miles	1.32	0.24	3.7	2.3, 6.0	<0.001
7–10 miles	1.35	0.34	3.9	2.0, 7.6	<0.001
10–12 miles	1.85	0.73	6.4	1.5, 26.7	0.011
12–15 miles	0.83	0.61	2.3	0.7, 7.6	0.179
>15 miles	1.03	0.61	2.8	0.8, 9.3	0.091
Not sure	–0.15	0.22	0.9	0.6, 1.3	0.497
**Average time spent exercising on a weekend day (n=5424)**					
Up to 30 min	Reference				
30 min–1 h	0.78	0.20	2.2	1.5, 3.3	<0.001
1–2 h	1.62	0.20	5.1	3.5, 7.4	<0.001
2–3 h	2.10	0.23	8.2	5.2, 12.8	<0.001
3–4 h	2.28	0.34	9.8	5.0, 19.0	<0.001
>4 h	1.33	0.35	3.8	1.9, 7.4	<0.001
**Average exercise distance covered on a weekend day (n=5423)**					
Up to 1 mile	Reference				
1–2 miles	0.60	0.21	1.8	1.2, 2.7	0.004
2–5 miles	1.83	0.19	6.2	4.3, 9.1	<0.001
5–7 miles	2.23	0.22	9.3	6.1, 14.2	<0.001

7–10 miles	2.28	0.26	9.7	5.8, 16.4	<0.001
10–12 miles	2.37	0.37	10.7	5.2, 22.3	<0.001
12–15 miles	2.44	0.53	11.5	4.0, 32.4	<0.001
>15 miles	1.52	0.36	4.6	2.2, 9.3	<0.001
Not sure	0.57	0.23	1.8	1.1, 2.8	0.011
**Availability of local off-road routes (n = 5256)**					
Very few	0.37	0.15	1.4	1.1, 2.0	0.017
Few	0.81	0.19	2.2	1.5, 3.3	<0.001
Moderate	0.79	0.19	2.2	1.5, 3.2	<0.001
Several	0.92	0.21	2.5	1.7, 3.8	<0.001
Spoilt for choice	Reference				
Don't know	-0.66	0.51	0.5	0.2, 1.4	0.200
**Distance to nearest off-road route (n = 5256)**					
Within 1 mile	Reference				
Between 1–2 miles	0.83	0.17	2.3	1.6, 3.2	<0.001
Between 2–5 miles	–0.01	0.16	1.0	0.7, 1.4	0.960
>5 miles	–0.13	0.19	0.9	0.6, 1.3	0.477
Don't know	–0.77	0.28	0.5	0.3, 0.8	0.006
**Flow of local off-road riding routes (n = 5256)**					
Very poorly connected	1.00	0.17	2.7	2.0, 3.8	<0.001
Poorly connected	1.40	0.19	4.0	2.8, 5.9	<0.001
Adequately connected	1.32	0.20	3.7	2.5, 5.5	<0.001
Well connected	1.48	0.25	4.4	2.7, 7.2	<0.001
Very well connnected	Reference				
Don't know	0.12	0.27	1.1	0.7, 1.9	0.664
**Horses exercised in-hand (n = 5426)**					
No	Reference				
Yes	–0.48	0.11	0.6	0.5, 0.8	<0.001
**Horses exercised by lungening (n = 5426)**					
No	Reference				
Yes	–0.15	0.11	0.9	0.7, 1.1	0.166
**Horses exercised by long-lining (n = 5426)**					
No	Reference				
Yes	–0.39	0.13	0.7	0.5, 0.9	0.002
**Horses exercised by leisure riding (n = 5426)**					
No	Reference				
Yes	1.56	0.14	4.8	3.6, 6.2	<0.001
**Horses exercised by ridden schooling/training (n = 5426)**					
No	Reference				
Yes	-0.06	0.12	0.9	0.7, 1.2	0.617
**Horses exercised by competitive riding (n = 5426)**					
No	Reference				
Yes	0.03	0.13	1.0	0.8, 1.3	0.844
**Horses exercised by carriage driving (n = 5426)**					
No	Reference				
Yes	0.61	0.33	1.8	1.0, 3.5	0.064
**Horses exercised by riding while leading a non-ridden horse (n = 5426)**					
No	Reference				
Yes	0.39	0.22	1.5	1.0, 2.3	0.077
**Horses exercised by riding while leading a ridden horse (n = 5426)**					
No	Reference				
Yes	0.77	0.34	2.2	1.1, 4.2	0.025
**Horses exercised by lead-rein (n = 5426)**					
No	Reference				
Yes	0.16	0.21	1.2	0.8, 1.8	0.447
**Equestrians' age category (n = 4842)**					

Under 18 years	0.62	0.49	1.9	0.7, 4.8	0.200
18–24 years	0.49	0.30	1.6	0.9, 2.9	0.098
25–34 years	0.60	0.26	1.8	1.1, 3.1	0.023
35–44 years	0.24	0.21	1.3	0.8, 1.9	0.254
45–54 years	0.38	0.20	1.5	1.0, 2.2	0.059
55–64 years	-0.03	0.19	1.0	0.7, 1.4	0.895
>64 years	Reference				
**Equestrian experience (n = 4846)**					
Up to 5 years	0.56	0.37	1.7	0.8, 3.6	0.132
6–10 years	0.13	0.25	1.1	0.7, 1.9	0.602
11–15 years	0.05	0.22	1.1	0.7, 1.6	0.819
16–20 years	0.28	0.21	1.3	0.9, 2.0	0.179
21–25 years	0.09	0.28	1.1	0.6, 1.9	0.751
26–30 years	0.35	0.21	1.4	0.9, 2.2	0.106
>30 years	Reference				

Table S3. Univariable logistic regression modeling to identify factors associated with higher odds of having had a road-related near-miss while using roads with a horse in the previous year.

Variable	Coefficient	Standard Error	Odds ratio (OR)	95% Confidence Interval (OR)	Wald *p*-Value
**Country (n = 5122)**					
Scotland	Reference				
England	0.49	0.10	1.6	1.3, 2.0	<0.001
Wales	0.59	0.13	1.8	1.4, 2.3	<0.001
Northern Ireland	0.46	0.30	1.6	0.9, 2.8	0.128
**Region of the UK (n = 4906)**					
Scotland	Reference				
East of England	0.20	0.12	1.2	1.0, 1.5	0.088
East Midlands	0.49	0.14	1.6	1.2, 2.1	<0.001
South East & London	0.39	0.12	1.5	1.2, 1.9	0.001
North East	0.60	0.23	1.8	1.7, 2.9	0.009
North West	0.95	0.15	2.6	1.9, 3.5	<0.001
Northern Ireland	0.46	0.30	1.6	0.9, 2.8	0.128
South West	0.57	0.12	1.8	1.4, 2.2	<0.001
Wales	0.59	0.13	1.8	1.4, 2.3	<0.001
West Midlands	0.55	0.15	1.7	1.3, 2.3	<0.001
Yorkshire & the Humber	0.83	0.17	2.3	1.6, 3.2	<0.001
**Frequency of road use (n = 5122)**					
Don't use them/infrequently (<once/week)	Reference				
1–2 days/week	0.83	0.09	2.3	1.9, 2.7	<0.001
2–3 days/week	1.06	0.10	2.9	2.4, 3.5	<0.001
3–4 days/week	1.19	0.11	3.3	2.7, 4.0	<0.001
4–5 days/week	1.19	0.12	3.3	2.6, 4.2	<0.001
5–6 days/week	1.18	0.15	3.3	2.4, 4.4	<0.001
6–7 days/week	1.38	0.13	4.0	3.1, 5.2	<0.001
**Distance to nearest off-road route (n = 5122)**					
Within 1 mile	Reference				
Between 1–2 miles	0.66	0.08	1.9	1.7, 2.2	<0.001
Between 2–5 miles	0.78	0.10	2.2	1.8, 2.7	<0.001
>5 miles	0.75	0.12	2.1	1.7, 2.7	<0.001
Don't know	0.04	0.21	1.0	0.7, 1.6	0.833
**Flow of local off-road riding routes (n = 5122)**					
Very poorly connected	1.50	0.12	4.5	3.5, 5.6	<0.001

Poorly connected	1.14	0.12	3.1	2.5, 3.9	<0.001
Adequately connected	0.81	0.12	2.3	1.8, 2.9	<0.001
Well connected	0.52	0.14	1.7	1.3, 2.2	<0.001
Very well connnected	Reference				
Don’t know	0.63	0.20	1.9	1.3, 2.8	0.002
**Walk horse in-hand on roads (n = 5122)**					
No	Reference				
Yes	0.12	0.07	1.1	1.0, 1.3	0.093
**Long-line horse on roads (n = 5122)**					
No	Reference				
Yes	0.56	0.13	1.7	0.4, 2.3	<0.001
**Ride on roads (n = 5122)**					
No	Reference				
Yes	0.94	0.09	2.6	2.1, 3.1	<0.001
**Carriage drive on roads (n = 5122)**					
No	Reference				
Yes	0.56	0.15	1.7	1.3, 2.4	<0.001
**Ride while leading a non-ridden horse on roads (n = 5122)**					
No	Reference				
Yes	0.43	0.11	1.5	1.2, 1.9	<0.001
**Ride while leading a ridden horse on roads (n = 5122)**					
No	Reference				
Yes	0.62	0.16	1.9	1.4, 2.5	<0.001
**Lead-rein on roads (n = 5122)**					
No	Reference				
Yes	0.43	0.12	1.5	1.2, 1.9	<0.001
**Level of anxiety when using roads with their horse (n = 5122)**					
Not at all anxious	Reference				
Slightly anxious	0.88	0.10	2.4	2.0, 2.9	<0.001
Moderately anxious	1.46	0.11	4.3	3.5, 5.3	<0.001
Extremely anxious	1.76	0.13	5.8	4.5, 7.4	<0.001
No longer use roads	–0.51	0.18	0.6	0.4, 0.9	<0.001
**Equestrians’ age category (n = 4842)**					
Under 18 years	0.02	0.22	1.0	0.7, 1.6	0.915
18–24 years	0.32	0.15	1.4	1.0, 1.9	0.032
25–34 years	0.44	0.13	1.6	1.2, 2.0	0.001
35–44 years	0.34	0.12	1.4	1.1, 1.8	0.004
45–54 years	0.34	0.11	1.4	1.1, 1.7	0.002
55–64 years	0.23	0.11	1.3	1.0, 1.6	0.036
>64 years	Reference				
**Equestrian experience (n = 4846)**					
Up to 5 years	Reference				
6–10 years	0.39	0.19	1.48	1.0, 2.2	0.043
11–15 years	0.42	0.18	1.52	1.1, 2.2	0.022
16–20 years	0.62	0.18	1.85	1.3, 2.6	<0.001
21–25 years	0.51	0.21	1.67	1.1, 2.5	0.012
26–30 years	0.63	0.18	1.88	1.3, 2.7	<0.001
>30 years	0.60	0.15	1.82	1.3, 2.5	<0.001

Table S4. Univariable logistic regression modeling to identify factors associated with higher odds of having had a road-related injury-causing incident (injury to equestrian and/or horse) while using roads with a horse in the previous year.

Variable	Coefficient	Standard Error	Odds ratio (OR)	95% Confidence Interval (OR)	Wald *p*-Value
**Country (n = 5122)**					
Scotland	Reference				
England	0.39	0.23	1.5	0.9, 2.3	0.094
Wales	0.62	0.27	1.9	1.1, 3.2	0.023
Northern Ireland	1.02	0.49	2.8	1.1, 7.2	0.036
**Region of the UK (n = 4906)**					
Scotland	Reference				
East of England	−0.07	0.29	0.9	0.5, 1.7	0.813
East Midlands	0.45	0.30	1.6	0.9, 2.8	0.135
South East & London	0.18	0.28	1.2	0.7, 2.1	0.535
North East	−0.14	0.56	0.9	0.3, 2.6	0.802
North West	0.64	0.29	1.9	1.1, 3.4	0.027
Northern Ireland	1.02	0.49	2.8	1.1, 7.2	0.036
South West	0.60	0.26	1.8	1.1, 3.1	0.024
Wales	0.62	0.27	1.9	1.1, 3.2	0.023
West Midlands	0.50	0.31	1.6	0.9, 3.0	0.109
Yorkshire & the Humber	0.84	0.31	2.3	1.3, 4.3	0.007
**Frequency of road use (n = 5122)**					
Don't use them/infrequently (<once/week)	Reference				
1–2 days/week	0.06	0.20	1.1	0.7, 1.6	0.745
2–3 days/week	−0.08	0.21	0.9	0.6, 1.4	0.722
3–4 days/week	−0.01	0.22	1.0	0.6, 1.5	0.957
4–5 days/week	0.30	0.23	1.4	0.9, 2.1	0.178
5–6 days/week	0.04	0.30	1.0	0.6, 1.8	0.901
6–7 days/week	0.67	0.22	1.9	1.3, 3.0	0.002
**Distance to nearest off-road route (n = 5122)**					
Within 1 mile	Reference				
Between 1–2 miles	0.63	0.14	1.9	1.4, 2.5	<0.001
Between 2–5 miles	0.73	0.17	2.1	1.5, 2.9	<0.001
>5 miles	0.73	0.20	2.1	1.4, 3.1	<0.001
Don't know	0.64	0.38	1.9	0.9, 4.0	0.093
**Flow of local off-road riding routes (n = 5122)**					
Very poorly connected	0.93	0.30	2.5	1.4, 4.5	0.002
Poorly connected	0.49	0.31	1.6	0.9, 3.0	0.112
Adequately connected	0.47	0.31	1.6	0.9, 3.0	0.133
Well connected	−0.12	0.37	0.9	0.4, 1.9	0.752
Very well connnected	Reference				
Don't know	−0.46	0.65	0.6	0.2, 2.2	0.477
**Walk horse in-hand on roads (n = 5122)**					
No	Reference				
Yes	0.14	0.13	1.1	0.9, 1.5	0.304
**Long-line horse on roads (n = 5122)**					
No	Reference				
Yes	0.17	0.22	1.2	0.8, 1.8	0.444
**Ride on roads (n = 5122)**					
No	Reference				
Yes	−0.08	0.18	0.9	0.6, 1.3	0.667
**Carriage drive on roads (n = 5122)**					
No	Reference				
Yes	0.46	0.23	1.6	1.0, 2.5	0.043

**Ride while leading a non-ridden horse on roads (n = 5122)**					
No	Reference				
Yes	0.42	0.18	1.5	1.1, 2.2	0.021
**Ride while leading a ridden horse on roads (n = 5122)**					
No	Reference				
Yes	0.79	0.20	2.2	1.5, 3.3	<0.001
**Lead-rein on roads (n = 5122)**					
No	Reference				
Yes	0.20	0.20	1.2	0.8, 1.8	0.300
**Level of anxiety when using roads with their horse (n = 5122)**					
Not at all anxious	Reference				
Slightly anxious	0.27	0.28	1.3	0.8, 2.2	0.334
Moderately anxious	0.84	0.27	2.3	1.4, 4.0	0.002
Extremely anxious	1.33	0.28	3.8	2.2, 6.5	<0.001
No longer use roads	0.58	0.41	1.8	0.8, 4.0	0.161
**Equestrians' age category (n = 4842)**					
Under 18 years	0.52	0.40	1.7	0.8, 3.7	0.198
18–24 years	0.30	0.30	1.3	0.7, 2.4	0.321
25–34 years	0.61	0.25	1.8	1.1, 3.0	0.017
35–44 years	0.10	0.25	1.1	0.7, 1.8	0.682
45–54 years	0.22	0.23	1.2	0.8, 2.0	0.349
55–64 years	0.001	0.24	1.0	0.6, 1.6	0.997
>64 years	Reference				
**Equestrian experience (n = 4846)**					
Up to 5 years	Reference				
6–10 years	0.30	0.40	1.4	0.6, 3.0	0.451
11–15 years	0.09	0.39	1.1	0.5, 2.4	0.814
16–20 years	0.04	0.38	1.0	0.5, 2.2	0.912
21–25 years	0.08	0.43	1.1	0.5, 2.5	0.853
26–30 years	0.16	0.37	1.2	0.6, 2.4	0.668
>30 years	0.13	0.33	1.1	0.6, 2.2	0.708
**Road-related near-miss in the previous year (n = 5122)**					
No	Reference				
Yes	1.96	0.23	7.1	4.5, 11.1	<0.001
